# Inflammation and INOCA: Can Fat Attenuation Indexing by Coronary CT Angiography Help Identify Coronary Inflammation?

**DOI:** 10.1016/j.jaccas.2024.103215

**Published:** 2025-02-12

**Authors:** Jenna J. Port, Brittany N. Weber, Madhavi Kadiyala

**Affiliations:** a CardioVascular Center, Division of Cardiology, Department of Medicine, Tufts Medical Center, Boston, Massachusetts, USA; b Heart and Vascular Center, Division of Cardiovascular Medicine, Department of Medicine, Brigham and Women’s Hospital, Harvard Medical School, Boston, Massachusetts, USA

**Keywords:** fat attenuation index, hypereosinophilic syndrome, INOCA, perivascular adipose tissue, vasospasm

## Abstract

Hypereosinophilic syndrome (HES) is an inflammatory condition that rarely affects the heart. A 24-year-old woman presented with non–ST-segment elevation myocardial infarction and peripheral eosinophilia. Coronary vasospasm without obstructive coronary artery disease (CAD) was noted on cardiac catheterization, and cardiac magnetic resonance showed resting perfusion defects with multiple punctate infarcts. She ultimately received a diagnosis of HES resulting in ischemia with nonobstructive coronary arteries (INOCA). The presence of coronary inflammation was suggested by a newer coronary computed tomography postprocessing tool called the fat attenuation index (FAI). The FAI has been shown to identify high-risk atherosclerotic CAD and associated inflammation. The role of the FAI in nonatherosclerotic CAD is unknown. This report describes the first case of an abnormal FAI in a young woman with HES and INOCA. Patients with HES may present with coronary artery inflammation that can result in INOCA. There may be a potential role for the FAI in identifying coronary inflammation in INOCA.

## Visual Summary

**Figure:**
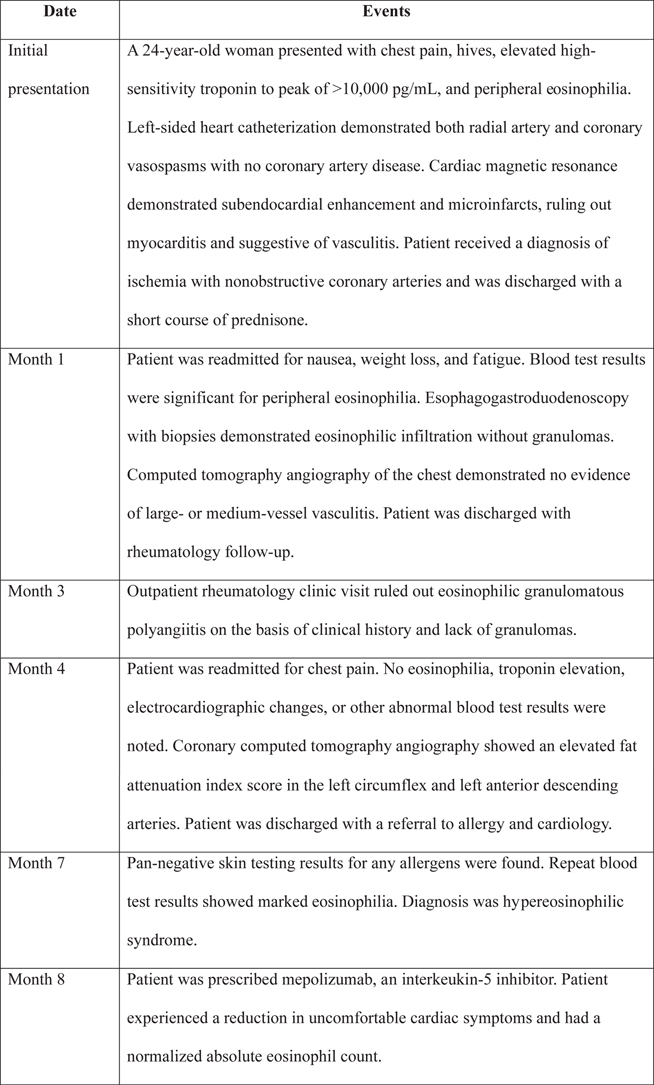

## History of Presentation

**Figure 1:**
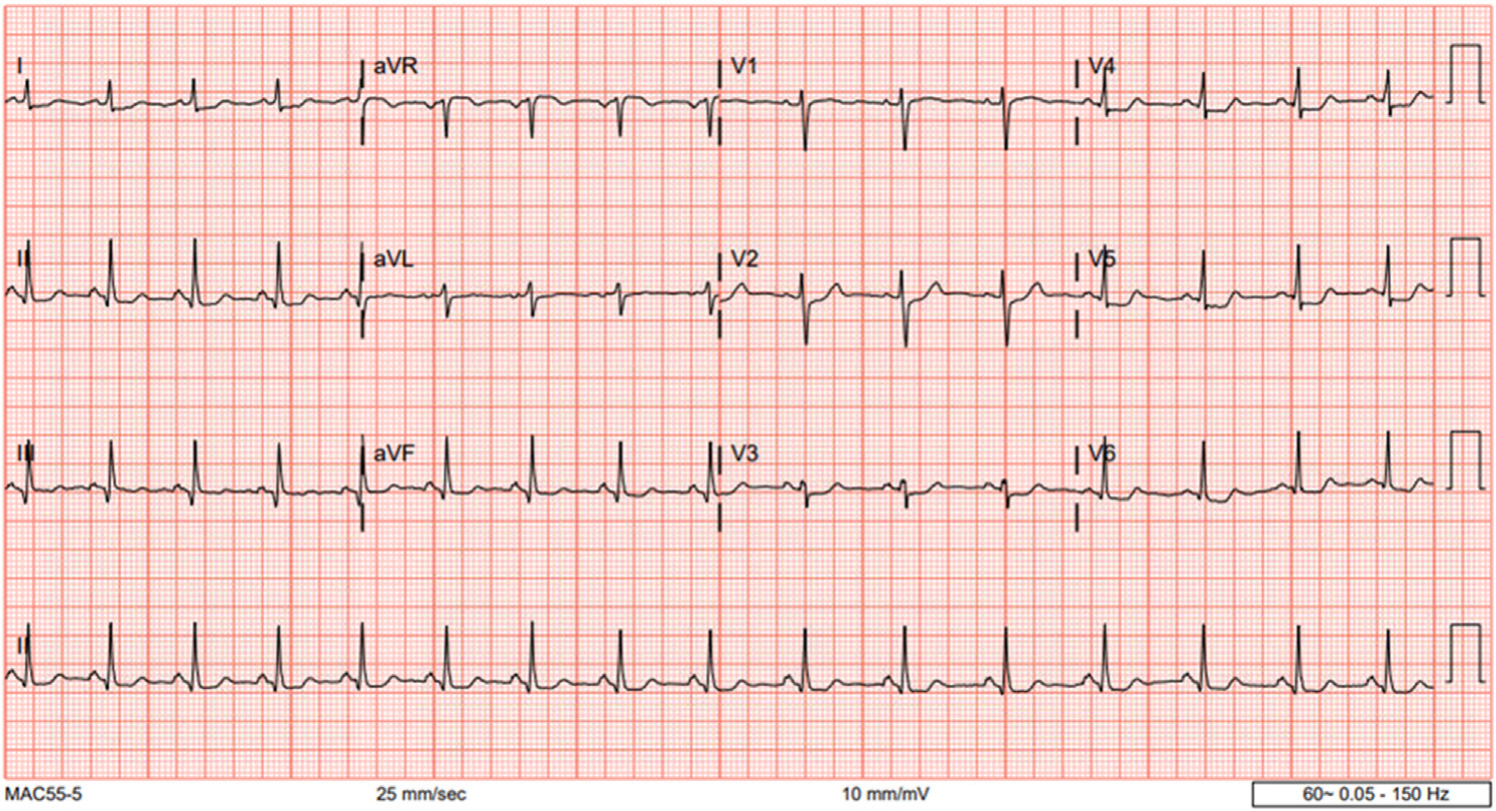
Initial Electrocardiogram of the Patient at the Time of Non–ST-Segment Elevation Myocardial Infarction Notable ST-segment depression is evident in leads V_3_ to V_6_.

A 24-year-old woman with a history of supraventricular tachycardia and recurrent admissions for chest pain presented to the emergency department (Charlton Memorial Hospital, Massachusetts, USA; patient was later transferred to Tufts Medical Center) with chest pain and a rash. On examination, she was afebrile with stable vital signs (heart rate, 93 beats/min; blood pressure, 122/87 mm Hg) and had diffuse urticaria. Blood test results revealed high-sensitivity troponin I to a peak of 10,725 pg/mL and marked eosinophilia (1,680 cells/μL). The electrocardiogram (ECG) demonstrated ST-segment depression in leads V_3_ to V_6_ (Figure 1). She was admitted urgently for coronary angiography.

**Take-Home Messages**

• FAI is a newer CT-based image processing technique that can detect coronary vascular inflammation in atherosclerotic CAD and may also play a role in nonatherosclerotic coronary artery inflammation.
• Further studies are required to investigate the use of FAI in patients with INOCA and underlying inflammatory disorders.

## Past Medical History

The patient has had multiple nonspecific symptoms, diagnosed at various times as generalized anxiety disorder, major depressive disorder, functional neurologic disorder, and conversion disorder. On chart review, there were other episodes of eosinophilia beginning 3 years earlier, with values of 550, 800, and 1,400 cells/μL, respectively.

## Differential Diagnosis

The differential diagnoses included distinguishing between myocardial ischemia and myocarditis. Causes of myocardial injury such as supply-demand mismatch, coronary vasospasm, coronary embolism, spontaneous coronary artery dissection, and vasculitis were considered. Given the eosinophilia, rare causes of ischemia such as vasculitis related to hypereosinophilic syndrome (HES) and allergic angina were also considered.

## Investigations

**Figure 2:**
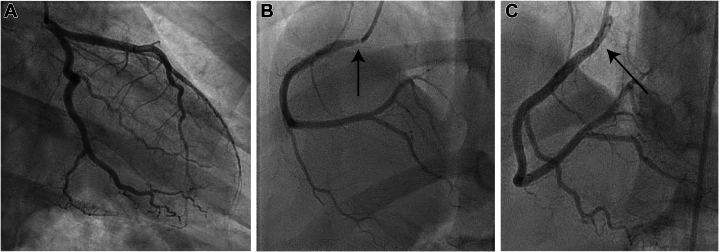
Coronary Angiography During the Initial Admission (A) Left-sided coronary circulation with no angiographic evidence of obstructive coronary artery disease. (B) Spasm of the right coronary artery was noted (arrow). (C) This spasm was alleviated (arrow) following intravenous nitroglycerin administration.

**Figure 3:**
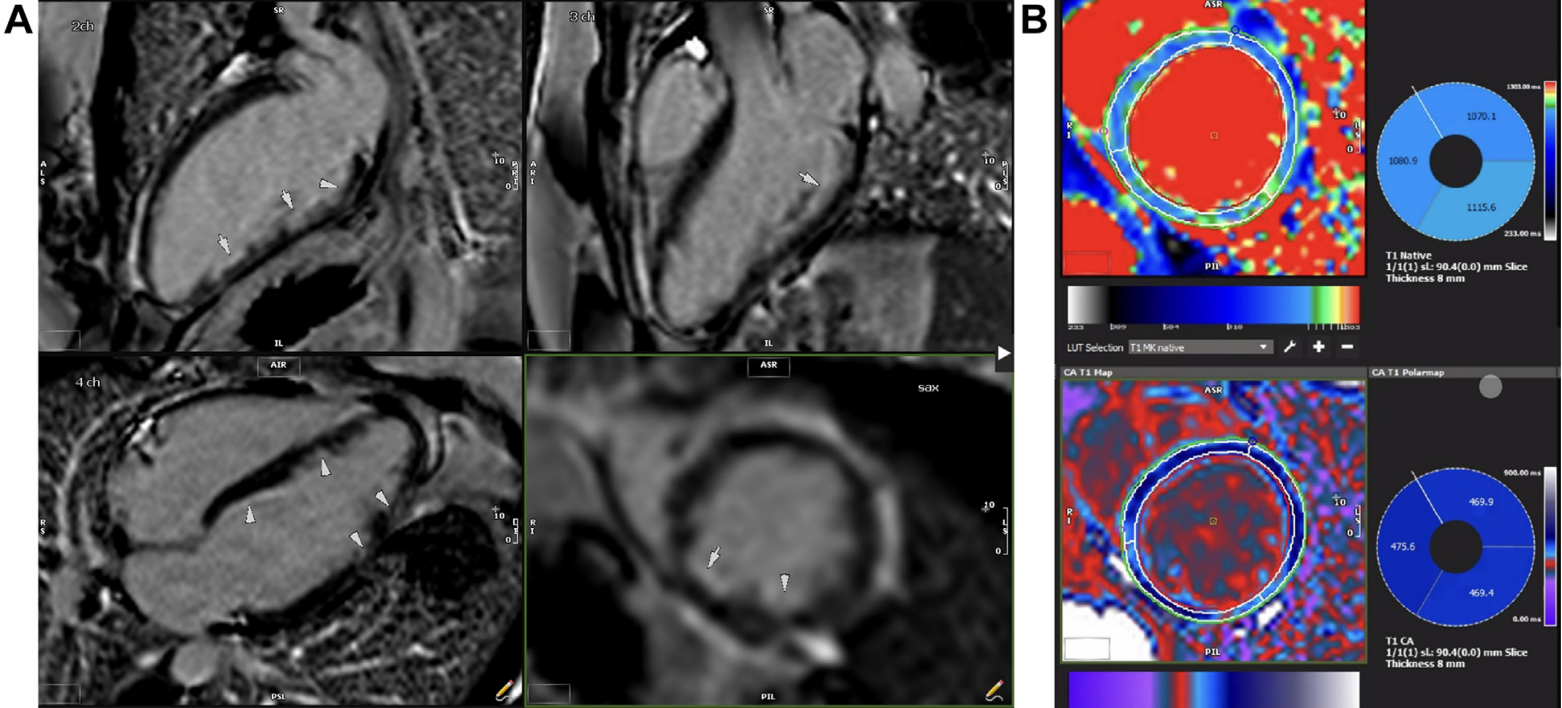
Late Gadolinium and T1 Mapping Images (A) Late gadolinium images demonstrate multiple punctate areas of late gadolinium enhancement (arrowheads). (B) T1 mapping with abnormal native T1 value in the lateral segment. ASR = anterior, superior, right; CA = contrast agent; PIL = posterior inferior lateral; RI = right inferior.

Coronary angiography demonstrated normal coronary vasculature without obstructive coronary artery disease (CAD). During catheterization, she developed a radial artery spasm as well as vasospasm of the right coronary artery, which resolved with sublingual nitroglycerin (Figures 2A to 2C). Myocarditis was considered likely, and cardiac magnetic resonance (CMR) was performed. CMR demonstrated no myocardial edema on T2-weighted imaging or subepicardial late gadolinium enhancement (LGE) to suggest myocarditis. However, subendocardial LGE in the basal septum to the midanteroseptum and inferoseptum was present (Figures 3A), along with scattered punctate areas of enhancement in the inferior and inferolateral walls, consistent with microinfarcts and concerning for ischemic injury (Video 1). Additionally, there were resting myocardial perfusion defects in the papillary muscles (Video 1), suggesting ischemia as the primary process rather than the subepicardial inflammation expected in myocarditis. The myocardial involvement was not in a major epicardial coronary distribution. Given the eosinophilia and the concern for vasculitis, she was treated empirically with prednisone (60 mg for 5 days), resulting in resolution of peripheral eosinophilia and improvement in symptoms. She received a diagnosis of unspecified eosinophilic vasculitis and was referred to rheumatology.

She presented again 2 months later with nausea, weight loss, and fatigue. She again had peripheral eosinophilia (780 cells/μL), which spontaneously resolved without steroids. Cardiovascular test results were notable for a normal electrocardiogram and a normal troponin level. Given her gastrointestinal symptoms, an endoscopy with duodenal biopsy was performed. Pathologic examination demonstrated eosinophilic infiltration without granulomas. Eosinophilic granulomatous polyangiitis, a small-vessel vasculitis, was considered, given the multisystem involvement. However, she did not meet the diagnostic criteria because of the absence of granulomas and the lack of other features such as chronic sinusitis, pulmonary infiltrates, neuropathy, palpable purpura, and nephritis.^1^ A computed tomography angiogram (CTA) of the chest lacked evidence of other large- or medium-vessel vasculitis. Systemic mastocytosis was considered; however, her tryptase level was normal. She was discharged on propranolol (10 mg 3 times a day) for symptomatic palpitations.

Three weeks after her second discharge, she presented again with chest pain, although this time her troponin and eosinophil values were both normal. Given the diagnostic uncertainty, a coronary CTA was performed, which revealed normal coronary artery origins and no coronary atherosclerosis (Supplemental Figure 1, Video 2). An investigational technique to assess coronary perivascular inflammation was applied to the coronary computed tomography (CT) images following acquisition. This technique, called the fat attenuation index (FAI), provides an inflammatory score relative to age- and gender-matched individuals.^2^ The left anterior descending and left circumflex arteries had a higher FAI score (83rd percentile and 75th percentile, respectively) compared with the matched control subjects, a finding suggestive of coronary vascular inflammation (Figure 3B). Allergic angina (Kounis syndrome), a rare disorder of coronary vasospasm and thrombosis related to an allergic trigger, was considered; however, this was ruled out with a comprehensive allergy assessment.

She once again experienced chest pain and fatigue and returned to her outpatient cardiologist, where blood test results demonstrated marked eosinophilia (5,069 cells/μL). Her cardiac studies to date were notable for variable vessel involvement over time. Although the initial ECG showed ischemic changes in the lateral leads, coronary vasospasm was noted in the right coronary artery during catheterization. CMR showed subendocardial LGE in the basal septum to midseptum and punctate areas of LGE in the inferior and lateral wall. On CTA, the FAI was elevated in the left anterior and left circumflex arteries. Such a fluctuating pattern and temporal variability in the coronary vascular involvement suggested a diffuse systemic inflammatory process. After extensive multidisciplinary discussion, we determined that the likely explanation for her recurrent episodes of chest pain was ischemia with nonobstructive coronary arteries (INOCA) secondary to HES causing perivascular coronary inflammation and vasospasm.^3^

## Management

Given the presumptive diagnosis of HES, she was started on mepolizumab, an interleukin-5 inhibitor, to reduce the number of circulating eosinophils.^4^ Mepolizumab has been shown to decrease the symptom burden of patients with HES, and in this patient, it will likely reduce future episodes of organ damage from eosinophilia.

## Outcome and Follow-Up

Immediately following the initiation of mepolizumab, the patient reported a significant reduction in the intensity of her intermittent chest discomfort. Her peripheral eosinophils immediately fell to a normal level of 163 cells/μL.

## Discussion

INOCA and myocardial infarction with nonobstructive coronary arteries (MINOCA) are generally considered to be less severe than obstructive atherosclerotic CAD, yet multiple studies iterate that MINOCA patients are at higher risk for cardiac events than those without known cardiovascular disease.^5^ Patients with MINOCA secondary to vasospasm have a significantly higher risk of cardiac death than do patients without spasm at 3-year follow-up.^6^ The cardiovascular prognosis varies on the origin of MINOCA; therefore, accurate diagnosis is a crucial step in management. There remains significant variability in how clinicians evaluate and treat patients with MINOCA because of the lack of guidelines and consensus on diagnostic and management approaches.

Inflammation is a well-established risk factor for atherosclerotic plaque rupture and myocardial injury.^7^ The LoDoCo-2 (Low dose Colchicine vs Placebo in patients with Chronic Coronary Disease) trial demonstrated that reduction of systemic inflammation significantly reduced major adverse cardiac events and improved clinical outcomes following myocardial infarction.^8^ Despite the known importance of vascular inflammation, there has been a scarcity of reliable biomarkers and imaging markers of coronary inflammatory burden. One potential imaging biomarker is the FAI, which can be derived from coronary CTA images. The FAI is a postacquisition image processing technique that can assess physiologic changes of perivascular adipose tissue in response to an inflammatory milieu.^2^ Studies have shown that identification of an increased FAI around the coronary arteries is strongly predictive of fatal cardiac events and nonfatal myocardial infarction (MI).^2^^,^^9^ In atherosclerotic CAD, the FAI and patient demographic data can be integrated into an FAI score to predict an individualized risk of cardiac mortality, nonfatal MI, and other cardiovascular events. An FAI score higher than the 75th percentile results in a 2.4-fold increase in relative risk for fatal cardiac events.^10^

**Figure 4:**
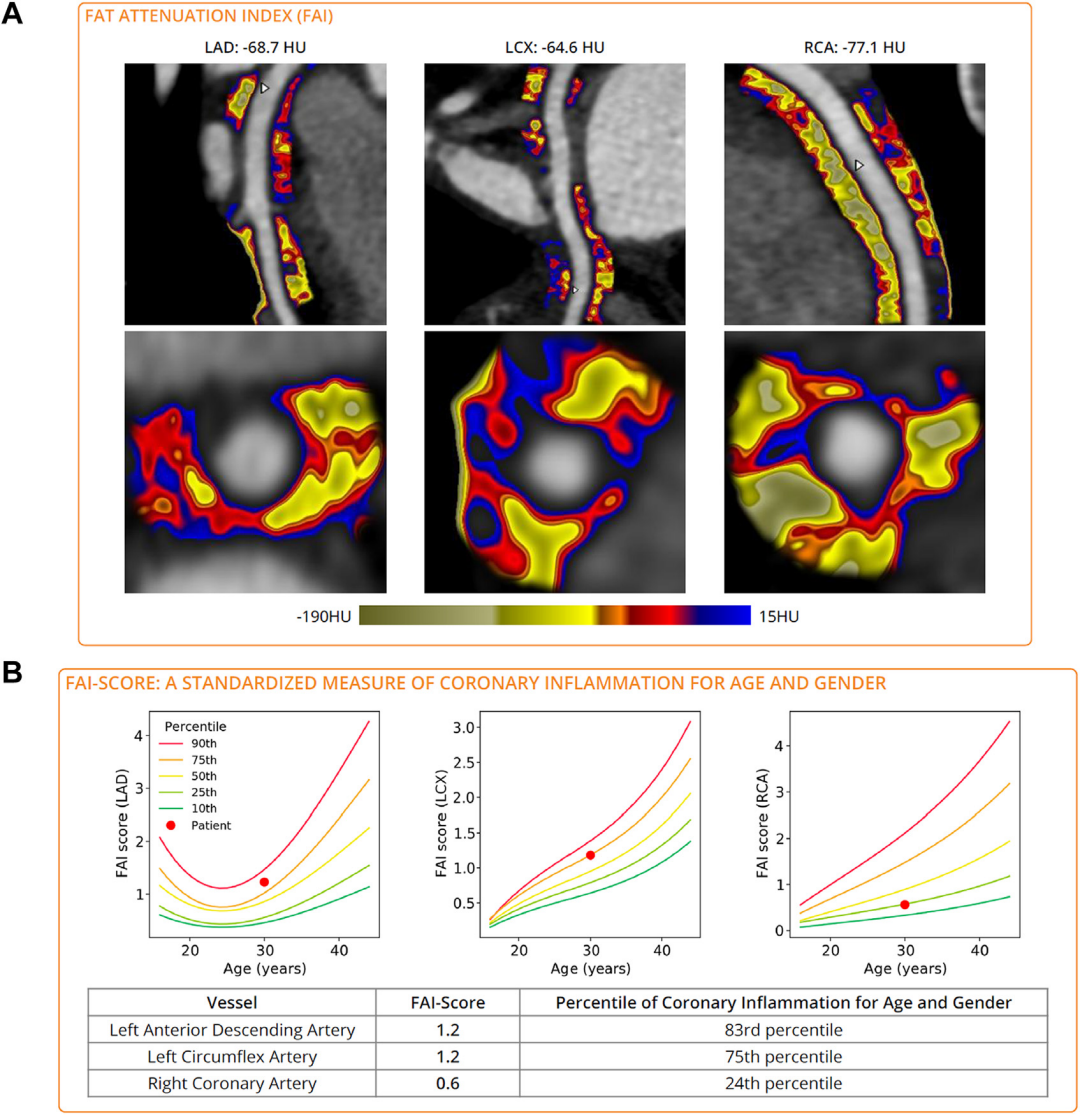
FAI of All 3 Coronary Arteries and Overall FAI Score (A) The fat attenuation index (FAI) as an unadjusted visual representation of inflammation along the 3 main coronary arteries. (B) The calculated FAI score of the left anterior descending (LAD) and left circumflex (LCX) arteries was 1.2, falling into the 83rd and 75th percentiles, respectively, among individuals of the same age and gender. The FAI score of the right coronary artery (RCA) was normal. Analysis was performed by Caristo Diagnostics and is not approved by the Food and Drug Administration.

Given the lower incidence of MINOCA and INOCA, there have been relatively few studies on the role of the FAI on patients with nonatherosclerotic vascular disease, such as vasospastic angina or vasculitis. The available studies note higher pericoronary inflammation in INOCA, a finding suggesting a potential role for FAI in patients with coronary inflammation but without atherosclerosis, such as this patient, who would be classified as “low risk” on the basis of current evidence. While acknowledging that the FAI score described earlier is validated only in patients with presumed atherosclerotic CAD disease and is still investigational in the United States, we assessed the FAI of the coronary arteries in this patient with INOCA. Corrected for a 30-year-old woman (data not yet available for subjects aged <30 years), her FAI score placed her in the 83rd percentile for future cardiovascular events (Figures 4A and 4B).

Patients who present with MINOCA or INOCA syndromes have a wide variety of causes, including coronary artery dissection, coronary artery embolism, coronary vasospasm, microvascular dysfunction, plaque disruption, vasculitis, and more. A subset of these patients has coronary inflammation driving the ischemia and myocardial damage. We hypothesize that our patient’s recurrent chest pain and myocardial injury resulted from episodes of perivascular inflammation driven by hypereosinophilia, triggering coronary vasospasm and ischemia. There is a growing body of literature on the role of treating coronary inflammation in CAD. FAI may have a role in detecting a high-risk subset of MINOCA and INOCA patients with underlying coronary inflammation. These patients may lack traditional risk factors yet continue to present with repeated hospitalizations and debilitating symptoms. Identification of perivascular inflammation by imaging techniques has the potential to stratify which patients may benefit from systemic anti-inflammatory agents. Further prospective studies are needed to investigate the role of FAI in patients with INOCA and systemic inflammatory disorders.

## Conclusions

FAI is a postacquisition CT processing technique that can detect perivascular inflammation in atherosclerotic CAD. Abnormal FAI has been linked to a higher risk of adverse cardiac events. The role of FAI in nonatherosclerotic causes of coronary inflammation, such as in patients with vasculitis or other autoimmune conditions, is not known. This case report, of abnormal FAI in a young woman with INOCA and HES, highlights the potential role of identifying coronary inflammation in patients with inflammatory disorders and INOCA.

**Visual Summary:** A Timeline of the Patient’s Presentation Through Diagnosis

Date	Events
Initial presentation	A 24-year-old woman presented with chest pain, hives, elevated high-sensitivity troponin to peak of >10,000 pg/mL, and peripheral eosinophilia. Left-sided heart catheterization demonstrated both radial artery and coronary vasospasms with no coronary artery disease. Cardiac magnetic resonance demonstrated subendocardial enhancement and microinfarcts, ruling out myocarditis and suggestive of vasculitis. Patient received a diagnosis of ischemia with nonobstructive coronary arteries and was discharged with a short course of prednisone.
Month 1	Patient was readmitted for nausea, weight loss, and fatigue. Blood test results were significant for peripheral eosinophilia. Esophagogastroduodenoscopy with biopsies demonstrated eosinophilic infiltration without granulomas. Computed tomography angiography of the chest demonstrated no evidence of large- or medium-vessel vasculitis. Patient was discharged with rheumatology follow-up.
Month 3	Outpatient rheumatology clinic visit ruled out eosinophilic granulomatous polyangiitis on the basis of clinical history and lack of granulomas.
Month 4	Patient was readmitted for chest pain. No eosinophilia, troponin elevation, electrocardiographic changes, or other abnormal blood test results were noted. Coronary computed tomography angiography showed an elevated fat attenuation index score in the left circumflex and left anterior descending arteries. Patient was discharged with a referral to allergy and cardiology.
Month 7	Pan-negative skin testing results for any allergens were found. Repeat blood test results showed marked eosinophilia. Diagnosis was hypereosinophilic syndrome.
Month 8	Patient was prescribed mepolizumab (300 mg once a month), an interkeukin-5 inhibitor. Patient experienced a reduction in uncomfortable cardiac symptoms and had a normalized absolute eosinophil count.

## Funding Support and Author Disclosures

This project has received no funding. Dr Weber serves as a consultant and on the scientific advisory board for Kiniska, Novo Nordisk and Bristol Myers Squibb; and has served on the scientific advisory board for Horizon. Dr Kadiyala has served as a consultant for HeartFlow. Dr Port has reported that she has no relationships relevant to the contents of this paper to disclose.

## Appendix

Video 1 Resting Perfusion Cardiac Magnetic Resonance First-pass gadolinium images of the left ventricle show prominent hypoperfusion in the papillary muscles in a noncoronary distribution. There were no perfusion defects noted in the coronary artery distribution.

Video 2 Coronary CTA Coronary CTA with normal coronary artery origins and without coronary atherosclerosis.
