# Supplementary material for: Inflammation and INOCA: Can Fat Attenuation Indexing by Coronary CT Angiography Help Identify Coronary Inflammation?
Source: JACC Case Rep. 2025 Feb 12;30(8):103215. doi: 10.1016/j.jaccas.2024.103215 (PMC12047009; doi:10.1016/j.jaccas.2024.103215)

**Supplemental Figure 1: Curved multiplanar reconstruction of the coronary arteries.** (A) Left circumflex artery (B) Right coronary artery (C) Left anterior descending coronary artery. The three main coronary arteries lacked any unusual characteristics, high-risk plaque features, or calcifications.


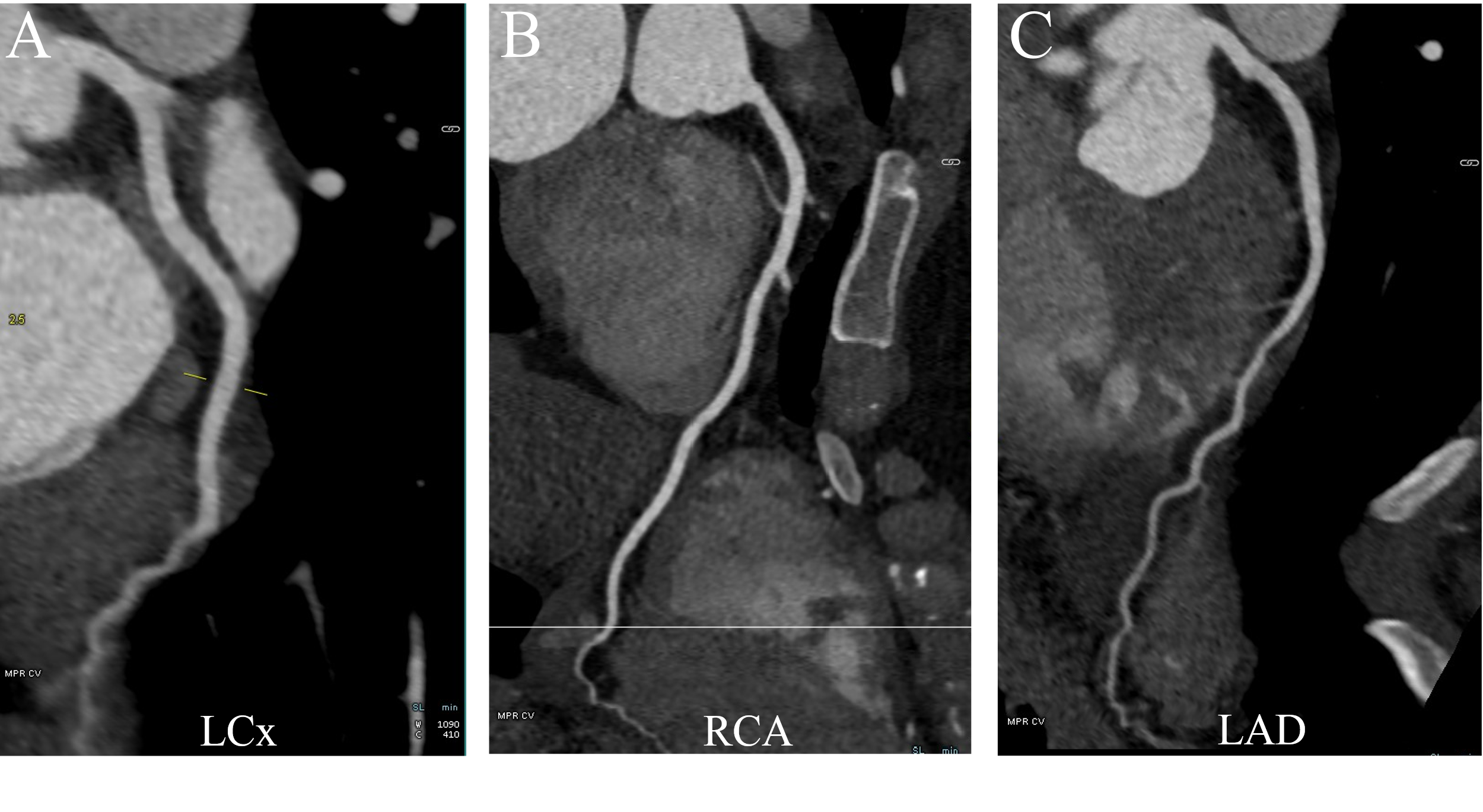

Supplement: Supplemental Figure 1 [file mmc3.docx]
